# Everyday environmental exposures and mid-life dietary and physical activity variations: E3 study protocol

**DOI:** 10.3389/fpubh.2025.1719726

**Published:** 2026-01-05

**Authors:** Ulf G. Bronas, Kiarri N. Kershaw, Jieqi Tu, Nathan Ryder, Jason Westra, Diego Redondo-Sáenz, Nathan Tintle

**Affiliations:** 1 Division of Research and Scholarship, Columbia University School of Nursing, New York, NY, United States; 2 Department of Preventive Medicine, Feinberg School of Medicine, Northwestern University, Chicago, IL, United States; 3 Department of Biostatistics, UIC School of Public Health, University of Illinois – Chicago, Chicago, IL, United States; 4 Department of Population Health Nursing Science, College of Nursing, University of Illinois – Chicago, Chicago, IL, United States

**Keywords:** activity space, exposome, mid-life, nutrition, physical activity

## Abstract

**Background:**

Poor dietary and physical activity (PA) behaviors increase risk for obesity, cancer, other chronic diseases, and contribute to disparities. Mid-life remains a vulnerable life stage, where obesity rates peak, and chronic diseases emerge. Neighborhood environments provide opportunities, barriers, and cues/triggers to engage in healthy or unhealthy behaviors. However, most research is limited to consideration of residential neighborhoods proximal to an individual’s primary residence and does not consider the environment’s role in within-person daily and momentary differences in behaviors. Furthermore, current research tends to ignore questions about for whom the environment matters and under what conditions.

**Objective:**

To address environmental exposures and provide a test of activity-space environmental explanations for between-and within-person diet and PA variations during mid-life. Our central hypothesis is that activity-space environmental exposures contribute to both between- and within-person variations in dietary and PA behaviors and influence these behaviors, more than residential-neighborhood environments alone. Additionally, we hypothesize that activity-space environmental exposures are more consequential for diet and PA when self-regulatory capacity, trait or state factors that affect a person’s ability to make efforts to regulate behavior are diminished.

**Methods:**

A total of 400 adults ages 40–64 were enrolled. We used a combination of geographically-explicit ecological momentary assessment (GEMA) methodologies: global positioning system (GPS) location tracking; smartphone-based mini-surveys of diet, PA, and state factors; and accelerometry, as well as three 24-h dietary recalls, anthropometric measurements, and questionnaires of trait and other factors. Routine, daily, and momentary activity-space measures were derived based on the spatial extent of their movement and duration of exposure. Residential and activity-space environment features were measured using GIS, including absolute and relative availability of healthful and unhealthful foods, walkability, recreational resource availability, and crime.

**Conclusion:**

This research employed a dynamic environmental exposure approach using GEMA to supply evidence on the environment’s role in between-and within-person variations in diet and PA during mid-life, a pivotal time, in a racially/ethnically diverse sample. As such it will contribute to the understanding of how environmental determinants of behaviors are studied, informing new targets for lifestyle and place-based interventions to improve health during mid-life.

## 1 Introduction

Dietary and physical activity (PA) behaviors either increase or decrease risk for obesity and chronic diseases and may contribute to health disparities (1–4). Mid-life is considered a transitional and vulnerable life stage where obesity rates peak and chronic disease emerge (5, 6). Understanding how the environment affects dietary and PA behaviors in mid-life is a critical public health challenge. The daily living environment provides opportunities (e.g., parks), barriers (e.g., crime), and cues or triggers (e.g., fast food restaurants) for behaviors that are healthy or unhealthy (7). Thus, neighborhood environments provides an important leverage points for large-scale population health impact (8–13). However, environment-behavior associations have been largely inconsistent (8, 11, 14).

Focus has been directed toward residential neighborhoods and does not take into account the environment where people in mid-life spend the majority of their time such as place of employment or where they spend their leisure time (activity spaces) (15, 16). Thus, mid-life adults encounter many different environments throughout their day that likely affect behaviors (17, 18). Relatively little is known about within-person daily behavior and how daily environmental interactions affect daily and within-moment behavior. Since behavior patterns are influenced and created by cumulative daily environmental interactions it is likely that behaviors vary from day to day, and even within the same day (19, 20). Additionally, the influence of environmental exposures on behavior likely vary depending on within-person traits and states (21, 22). Thus, there is a clear need to identify the multiple roles the environment affects diet and PA. The purpose of this study was to identify environmental exposures in a non-ubiquitous way and define activity-space environment(s) that explain between-and within-person diet and PA variations during mid-life. This manuscript details the protocol employed to identify between-and within-person variations that occur in mid-life based on both neighborhood level and daily life-exposures outside of residential neighborhoods.

**Figure 1:**
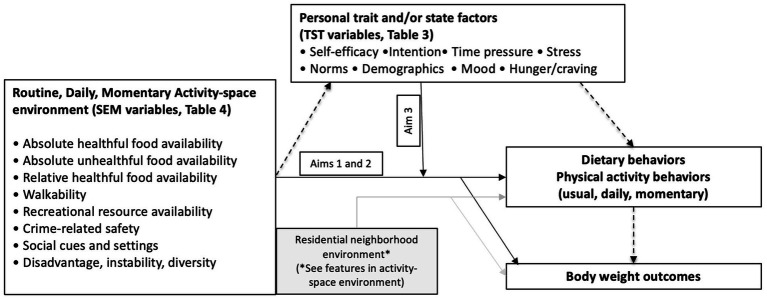
Conceptual model.

The central hypothesis that guides this study is that daily environmental activity-space exposures affect both between- and within-person variations in dietary and PA behaviors and to a greater degree than residential neighborhood environment alone. We also draw on tenets from the Temporal Self-Regulation Theory (TST) (23), hypothesizing that daily activity-space environmental exposures are more influential for diet and PA behavior when self-regulatory capacity, trait or state factors, affect the individual’s ability to make an effort to self-regulate behavior is reduced (24). The conceptual model (Figure 1) integrates principles from both the social ecological model, which posits that multilevel environmental factors interact to affect behavior, and the TST, which emphasizes self-regulatory capacity, trait and state factors. Thus, we will not only examine why some mid-life adults are more physically active or have a healthier diet (or are more likely to meet guidelines) than others (between-person differences), but also why they are more active or eat healthier (or meet guidelines) on some days than on others (within-person differences). This approach will allow us to determine the overall impact of how the environment where individuals conduct their activities and spend their time in daily life affects daily diet and PA behavior. We also include momentary activity-space environments to determine within-person daily and in-the-moment differences in behaviors. Finally, using TST constructs assessed via event- and signal-contingent ecological momentary assessment (EMA), we will determine how the effects of environmental exposures on diet and PA vary as a function of personal traits (long-standing characteristics) and states (temporary conditions or situations). This will allow us to determine for whom and under what conditions, environmental contexts are most influential. Guided by the conceptual model the study assessed the following aims.

The specific aims were to:

1. Determine the contributions of absolute and relative availability of healthy and unhealthy food in the activity space to between-person and within-person daily (and in-the-moment) differences in dietary behaviors.
2. Determine contributions of activity-space walkability, recreational resource availability, and safety to between-person and within-person daily (and in-the-moment) differences in PA behaviors.
3. Determine if sex, age, and factors in the TST (personal trait and state factors of self-regulatory capacity and behavioral prepotency) moderate activity-space environment-(1) diet and –(2) PA associations (Aims 1 and 2).
4. Explore whether sex, age, and factors in the TST (personal trait and state factors of self-regulatory capacity and behavioral prepotency) moderate between-person and within-person associations between activity-space environmental exposures and dietary and PA behaviors.

## 2 Methods and analysis

### 2.1 Design

This was a cross-sectional study designed to investigate the impact of neighborhood and activity-space environment on diet and PA among mid-life adults in Cook County, Illinois, with all data collection taking place within a single three-week time period, no intervention, but with in-depth, temporal measurements throughout the 3-week period to characterize within-person behavioral fluctuation, and not to infer causal temporal sequences. This study was approved by the University of Illinois Chicago Institutional Review Board and informed consent (e-consent) was obtained prior to study procedures.

Data collection for eligible individuals consisted of three phases: baseline visit, three-week study period, and follow-up visit. The entirety of the study was completed remotely. Interested participants first underwent a recruitment call with a staff member to learn about what the study entails and ensure eligibility. Study participants had a remote baseline visit where questionnaires were completed and set up for all components of data collection (24-h dietary recall, GEMA mini-surveys, accelerometer, GPS). After 21 days, participants underwent a remote follow-up visit for final questionnaires and a review of completed study components.

### 2.2 Selection and treatment of subjects

Individuals eligible for this study met the following criteria: (1) self-reported as NH White, NH Black or Hispanic/Latino; (2) ages of 40 and 64; (3) resided in Cook County; (4) had access to a smartphone; (5) was able to provide informed consent. Exclusion criteria included: (1) unable to speak English or Spanish (2) unable to read English; (2) not independently mobile or homebound. In addition, other racial/ethnic groups (which includes those who self-report as Asian, American Indian, Alaska Native, Native Hawaiian, and Other Pacific Islander) were excluded because of small sample sizes available in Cook County, IL.

To facilitate participant recruitment and retention, we hired and trained a racially/ethnically diverse team of research staff. Recruitment strategies included: (1) distribution of flyers targeting potential participants at community sites throughout the county; (2) distribution of recruitment messages through a mailing campaign for residents throughout the county; (3) public transit ads on subway lines serving communities with a high proportion of NH White, NH Black (Blue Line, Red Line), and Hispanic/Latino (Pink Line) populations; (4) outreach through a community consultant within the northern region of the county; (5) translation of study materials and surveys into Spanish.

We screened 1,307 participants for inclusion, 686 of whom met an exclusion criterion and 7 were uninterested. Of the 614 that met inclusion criteria and were interested, 204 did not complete baseline assessment and 10 withdrew within 5 days of baseline assessment. We therefore enrolled a sample of 400 participants in the study. Of the enrolled participants, the majority were female (58.0%, *n* = 232) and NH Black (37.5%, *n* = 150), consistent with Cook county demographics with a majority Female and a majority Black population (25). Additional details on demographics can be found in Table 1. Our primary referral sources were subway advertisement and word of mouth, which included 374 (61%) and 75 (12%) of eligible participants. Community organizers and advertisement accounted for <4% of the sample. The mean walk score was 74.2 (17.6).

**Table 1:** Demographic characteristics.

Characteristics	Mean/N (SD/%)
Mean age in years (SD)	50.6 (7.3)
Education	
Less than high school (*n*, %)	13 (3.2)
High school (*n*, %)	119 (29.8)
College: 2–4 years (*n*, %)	138 (34.5)
College: 5 + years (*n*, %)	130 (32.5)
Marital status	
Never married (*n*, %)	138 (34.5)
Married (*n*, %)	148 (37.0)
Divorced/Separated (*n*, %)	103 (25.8)
Widowed (*n*, %)	7 (1.8)
No answer (*n*, %)	4 (1.0)

### 2.3 Interventional methods

#### 2.3.1 Devices and data collection methods

Participants received an accelerometer (ActiGraph wGT3X-BT) to measure PA and sedentary time for 21 days. The data was collected in 1-min epochs. Participants were instructed to wear the device on their waist with a waistband throughout the day, except when bathing, swimming, or sleeping. A wear time of ≥10 h was defined as a valid day of accelerometer monitoring. Participants also receive a GPS device (Qstarz BT-Q1000XT GPS Data Logger) which records locations where participants conduct activity and travels. The data is collected in 1-min epochs. The GPS device was attached to a waistband alongside the accelerometer which participants are instructed to wear for 21 days. A wear time of ≥10 h was defined as a valid day of GPS tracking. GPS points will be used to construct routine and daily activity-space measures using ArcGIS. Time-stamped recordings from the accelerometer were linked with the GPS data using the AGSPR package in R which simultaneously cleans, imputes and harmonizes data at the minute epoch level (26). Across the 400 recruited individuals, 25 did not wear the GPS and 28 returned it with unusable data, leaving an N of 347 individuals with some usable GPS data. Valid mean days for GPS and EMA were 18.4 (4.6) and 10.7 (4.4), respectively. The study was designed to be well-powered (80%) to detect an effect size of 0.15 with a sample size of *N* = 350 for primary hypotheses.

In addition to the GPS device, a smartphone data collection app (MetricWire Inc.) was used to collect ecological momentary assessment (EMA) mini-surveys of behaviors and state factors. We used a combination of event- and signal-contingent sampling for the EMA mini-surveys (27). For event sampling, participants were asked to complete a mini-survey within 15 min of a certain behavior (e.g., eating or drinking, PA, walking for ≥10 min). This allowed signal-contingent sampling to enable a better understanding of personal and perceived environmental factors at these specific times. Participants were asked to complete 2 additional mini-surveys per day, for which they received a random prompt at random times. These random surveys were available for 60 min after an initial prompt with reminder prompts every 15 min. Completing ≥5 EMA mini-surveys a day was defined as a valid day of survey completion.

Self-reported data was collected during recruitment, baseline, and follow-up visits via REDCap. During recruitment, basic demographic information, and height and weight measurements were collected. During baseline, a 24-h dietary recall survey via a web-based tool [Automated Self-Administered 24-h Dietary Assessment Tool (ASA24®)] was completed. In addition, a baseline questionnaire was administered to collect data on neighborhood characteristics, diet, PA, wellbeing, health, annual household income and other demographics. During the follow-up, an activity space questionnaire was administered to collect data on locations participants most frequently visited during the study period. In addition, a follow-up questionnaire was completed to further collect information on health behaviors, substance use, diet, and psychosocial factors.

#### 2.3.2 Compliance

Staff followed up with participants regularly to answer questions and to ensure they did not have issues with device use or survey completion. Daily monitoring of participants’ mini-survey and GPS adherence using the secure MetricWire web platform ensured compliance with survey guidelines. In addition, staff recorded the number of completed 24-h dietary recalls at the end of participants’ 14-day survey period. Participants with remaining recalls were notified and asked to complete outstanding recalls. During the follow-up visits, participants who had not completed outstanding recalls were given a final opportunity to complete the remaining recalls.

#### 2.3.3 Schedule of events

Participants were screened for eligibility, comprehension and technological requirements during the recruitment call. This call includes an e-consent form, collection of basic demographic information and anthropometric measurements. During the baseline visit, we conducted several questionnaires and training on navigating the 24-h dietary recall survey, study devices, and EMA mini-surveys. The three-week study period comprised GPS tracking, accelerometry, EMA mini-surveys, and 24-h dietary recalls. We collected 3 weeks of GPS data to ensure that captured enough valid GPS days, as well as representation of weekend days to estimate participants’ routine activity space. We limited the EMA mini-surveys to the first 2 weeks to minimize participant burden and because 2 weeks provide ample within-person variation and statistical power. For the first 2 weeks, participants completed EMA mini-surveys using their personal smartphone with Metricwire’s mobile EMA application, available for both iOS/Apple and Android devices. We used a combination of event- and signal-contingent sampling for the EMA mini-surveys (27). For event sampling, participants were asked to complete a mini-survey within 15 min of (a) an eating/drinking episode, (b) doing PA/exercise/sports, and (c) walking continuously for ≥10 min (28). This allowed participants to report on behaviors, while minimizing recall bias in reporting state factors (e.g., mood, perceived environment) just prior to diet and PA events. In addition, signal-contingent sampling is used to understand personal and perceived environmental factors at times other than those co-occurring with diet or PA events. Specifically, participants were asked to complete 2 additional mini-surveys per day, for which they received a random prompt at random times. If one of these random prompts coincided with a diet or PA event, the EMA application directed them to an event survey. Thus, on a typical day, a participant completed 4 eating/drinking surveys (3 meals, 1 snack) and 2 random surveys. If PA, a participant also completed 1 (or more) daily surveys for PA or walking events. However, the number of surveys per day varied across participants and by day.

Staff contacted (text, email, phone) participants regularly to answer questions and encourage adherence. During the baseline visit, staff conduct a 24-h dietary recall, with the goal of obtaining one additional recall on a weekday and one recall on a weekend day. Staff monitored participants’ mini- survey (and GPS) adherence daily using the secure Metricwire web platform and contacted participants when they did not complete at least five daily EMA mini-surveys and/or the GPS data do not show movement.

#### 2.3.4 Measures

Table 2 outlines the primary outcomes: added sugars, fruit/vegetables, moderate-intensity PA, and walking. Our secondary outcomes included dietary (e.g., whole grains, fiber, saturated fat, overall diet quality via Healthy Eating Index-2015, and away-from-home restaurant intake) and PA (total activity counts, sedentary time, light PA, vigorous PA) behaviors (28–30).

**Table 2:** Measures of primary dietary and pa behavior outcomes: usual, daily, and momentary.

Behavior	Usual	Daily	Momentary
Added sugar	Mean daily intake, tsp	Daily intake, tsp	Intake of 5 sugary foods and 5 sugary drinks
	<10% mean daily calories from added sugar (3 24-h recalls)	<10% of daily calories from added sugar (24-h dietary recall)	
		Daily intake frequency of 5 sugary foods and 5 sugary drinks	
Fruit and vegetable intake	Mean daily intake, cup equivalents	Daily intake, cup equivalents	Intake of fruit, salad, and other vegetables
	≥5 mean daily cup eq. (3 24-h recalls)	>5 daily cup eq. (24-h dietary recalls)	
		Daily intake frequency of fruit, salad, and other vegetables	
Moderate PA	Mean weekly minutes (>2020 activity counts per minute)	Daily minutes	Minutes (Actigraph and adapted Godin)
	≥150 weekly minutes (Actigraph)	≥30 daily minutes (Actigraph and adapted Godin)	
Walking	Weekly minutes walking (1) for transport and (2) for recreation	Daily minutes walking (≥10 consecutive minutes) for transport and recreation	Walking for ≥10 consecutive minutes (1) for transport and (2) for recreation
	≥8,000 mean daily steps	≥8,000 daily steps (Actigraph)	

*Based on first 2 weeks.

Table 3 provides an overview of TST constructs; personal trait and state measures of self-regulatory capacity and behavioral prepotency (internal drives, norms) as well as demographics. All “state” measures were administered in the event- and signal-contingent EMA mini-surveys.

**Table 3:** Personal trait and state measures and timing.

Construct	Measure	Timing
Intention	Intentions for Moderate-to-Vigorous Physical Activity, fruit and vegetable intake, and discretionary food intake	Baseline
Self-efficacy	Self-efficacy for healthful eating, fruit and vegetable intake, and physical activity	Baseline
Mood states	Positive affect and negative affect	EMA
	Energetic arousal (tired, no energy) (Short Mood Scale)	EMA
Stress/Stress exposure	Daily hassles (Daily Inventory of Stressful Experiences – EMA version)	EMA
	“How stressed are you?” on Likert scale	EMA
	Acute life events and chronic difficulties (Stress and Adversity Inventory); Discrimination; Perceived stress scale	Baseline
	“Do you feel rushed and pressed for time?” on Likert scale	EMA
Internal drives	Food cravings (Desire to Eat Subscale, State General Food Cravings)	EMA
	Hunger and satiety visual analog scales	EMA
	Unhealthful eating, inactivity, and weight loss norms	Baseline
Demographics and others	Sex and gender; Age; Race and ethnicity; Socioeconomic status (e.g., annual household income, educational attainment, home ownership, employment, occupation); Auto ownership; Transport mode; Household composition; Marital status; Generational status and years in U. S.; Acculturation; Chronic health conditions; Menopausal status (females); Neighborhood preferences	Baseline

#### 2.3.5 Exposures

We constructed a rich geodatabase of environmental features using ArcGIS 10.6 based on both public and proprietary data for the Chicago metropolitan area was created. Table 4 provides an overview of SEM multilevel environmental constructs; environmental measures constructed for each participant’s routine, daily, and momentary activity spaces as well as their residential neighborhood.

**Table 4:** Measures of environmental exposures in activity space (AS) and residential neighborhood (RN).

Construct	Measure	AS	RN
Absolute healthful food availability	Densities of (1) Supermarkets and large grocery stores, (2) Farmers’ markets, (3) General merchandize store (e.g., Walmart, Target) (Commercial sources, government lists)	X	X
	+Perceived availability checklist: (1) Supermarkets and large grocery stores, (2) Farmer’s markets, (3) General merchandize store (e.g., Walmart, Target) (Visible or <5 min walk)	X	
	+Perceived availability checklist: (1) Fruits, (2) Vegetables (Visible and accessible)	X	
Absolute unhealthful food availability	Densities of (1) Convenience stores, (2) Fast food restaurants (Commercial sources, government lists)	X	X
	+Perceived availability checklist: (1) Convenience stores, (2) Fast food restaurant (Visible, <5 min walk)	X	
	+Perceived availability checklist: (1) Salty snacks, (2) Sweets (e.g., cookies), (3) Sugar-sweetened beverages (e.g., soda) (Visible and accessible)	X	
Relative food availability	Indices/ratios: (1) Number of supermarkets & large grocery stores to all retail food outlets including fast food restaurants and convenience stores, (2) Number fruits/vegetables to all assessed foods	X	X
Walkability	Index: (a) Land use (e.g., % commercial land use) and land use mix (CMAP Land Use Inventory and City of Chicago), (b) Street connectivity (e.g., % 4-way+ intersections) (StreetMapPro), (c) Residential density (ACS), (d) Public transit stop density (CTA, Metra, Pace)	X	X
	+Perceived – index: (a) Stores are within easy walking distance, (b) There are many places to go within walking distance, (c) It is easy to walk to a transit stop (Likert scale), adapted NEWS	X	
Recreational resource availability	Densities of (1) Parks (government listings), (2) Commercial fitness facilities/gyms (InfoUSA, Dun & Bradstreet), (3) Public recreation centers (government lists), (4) Walking/biking path or trail (government lists), (5) Bike lanes and paths (governmental lists), each as separate measures	X	X
	+Perceived availability: (1) Park, (2) Gym, (3) Walking or biking path/trail/lane (Visible,<5 min walk)	X	
	Greenness: Normalized difference vegetation index (satellite imagery), adjust. or interact. w/season	X	X
Crime-related safety	Index: Densities of violent crime: (a) homicide, (b) robbery, (c) criminal sexual assault, (d) aggravated assault (City of Chicago, Uniform Crime Report)	X	X
	+“How safe are you right now?” (Likert scale)	X	
Social setting/Cues	+Who with while eating or doing PA/exercise/sports (e.g., family, friend, alone)	X	
	+See someone else (1) Eating or drinking, (2) Exercising	X	
Behavioral settings	Place where obtained food/beverage (e.g., home, restaurant, vending machine), place where ate/drank (e.g., home, restaurant, car, outdoors)	X	
	Place where engaged in PA/exercise/sports (e.g., home, park, gym, streets near home)	X	
	Place where engaged in walking (e.g., home, park, gym, streets near home)	X	
Demographics	Economic disadvantage scale (poverty rate, unemployment rate, % female headed households, % cash assistance); (2) Residential instability scale (% residents who moved in past year; % renter occupied housing); (3) Racial diversity (relative portions of NH Blacks, NH Whites, Hispanic/Latino, others)	X	X

### 2.4 Data analysis

As refusals are anticipated for some questionnaire items, depending on missing data rates, missing patterns and missing values, we will ignore missing data, impute using regression models or use multiple imputations. We will construct measures separately, before combining and creating analytic datasets. Because environmental effects may not be linear, we will create continuous and categorical environment variables representing different levels (e.g., high, medium, low fast food restaurant density based on tertiles of the distribution). For each variable, we will examine descriptive statistics, frequency distributions, and graphical displays (e.g., box plots). For any continuous outcome variable that does not conform to a normal distribution, we will explore the use of transformations (e.g., logarithm). We will also examine patterns of association between variables and test for multicollinearity; we will consider indices or use of latent class analysis if multicollinearity is found in environmental variables. Reductions in dimensionality will allow us to minimize the need for multiple testing corrections, while still allow us to follow-up statistically significant relationships on all contributing individual measures to better understand patterns of association.

Because different levels and amounts of both the environmental exposures and dietary and PA behaviors are relevant, forms of variables implied in our hypotheses are examples. We will test both continuous and binary versions of the diet and PA outcomes (e.g., whether dietary and PA guidelines are met) (14, 15); thus, employing linear and logistic regression models. We will investigate usual behavior regressed on routine AS environmental measures, controlling for personal characteristics using separate models for each activity space and model with all relevant activity space variables together. Interactions between activity space and residential neighborhood environments will be tested. Additionally, EMA data (mini-surveys, accelerometry), 24-h dietary recall data, and daily/momentary environmental measures derived from GPS or EMA mini-surveys will be used for testing within-person hypotheses, allowing us to treat EMA data as time-varying measures. EMA reports will be used for construction of daily levels of perceived environmental exposures and personal state factors. A hierarchical structure with 2 weeks of daily observations clustered within participants will be created and we will explore first-order autoregressive structures between daily responses.

Analyses will include pre-specified consideration of potential sex, age, and race/ethnicity interactions. We will use interaction terms to test for potential differential relationships by these demographic variables, fitting strata specific estimates when we identify statistical evidence of differential relationships. Furthermore, multiple models will be fit for each hypothesis with different sets of covariates (a) unadjusted, (b) demographic adjusted (sex, age, race/ethnicity, ses), (c) demographic + medical history adjusted + seasonality adjustment.

We will also conduct a series of sensitivity analyses to ensure robustness of findings. First, we will utilize self-reported transportation and recruiting mode (e.g., subway ad, etc.) to mode to characterize the sample, and then fit models on subsamples of the data to ensure that findings hold true across individuals regardless of standard modes of transportation (e.g., car, bus, subway, walking). Second, while primary analyses will consider only an individual’s valid wear days (at least 10 h of wear time), and across 21 consecutive days of wear, we recognize that not all individuals may achieve this goal. Thus, we will conduct sensitivity analyses to ensure robustness of findings by considering (a) less stringent criteria for a valid day (e.g., 5 h) and (b) all valid days for a participant. We will also consider auto-regressive structures by units other than days (e.g., hours). When sensitivity analyses identify non-robust results and/or differences by groups, these will be explored further, contextualized and noted as limitations of the primary findings.

## 3 Discussion

This study contributes to a much-needed shift in how environmental determinants of behaviors are conceptualized and studied, and fill a significant knowledge gap in mid-life adults, positioning it to make a lasting impact on the field. By employing a dynamic environmental exposure approach using GEMA, this study will provide evidence regarding the environment’s role in between- and within-person variations in diet and PA behavior during mid-life, a pivotal time, in a racially/ethnically diverse sample. In addition, this study is among the first to integrate three distinct activity monitoring and geospatial devices for assessment of real-time environmental exposures, activity levels via accelerometers, and self-regulatory capacity, trait or state factors via EMA smartphone surveys. A sole focus on residential neighborhoods obscures spatial and temporal dimensions of behavior (i.e., residential neighborhoods generally do not capture residents’ mobility/movement) (17). The home generally serves as an anchor of a more expansive activity space, or area where a person conducts activities and spends time including workplaces, schools, and places for socialization and the routes a person travels between locations. Activity spaces are particularly relevant for mid-life adults who typically have higher participation in the labor force, and time spent at work. Additionally, family and other social obligations frequently take mid-life adults outside their residential neighborhood.

For many in mid-life, physical limitations and resource constraints have not yet hampered mobility, although they may become more dependent on the residential neighborhood as they approach late adulthood. This study will provide insight into the impact that supportive activity spaces have on diet and PA behavior. Having supportive activity spaces and residential neighborhoods may lead to healthier behaviors than having a supportive environment in only one context. Usual diet and PA behaviors result from the accumulation of daily and in-the-moment decisions about whether/what to eat/drink and how physically active to be as individuals navigate their daily environment. Much of the variation observed in diet and PA behaviors likely reflects within-person fluctuation, shaped by variation in environmental exposures. Thus, changing environmental conditions likely affect a person’s ability to self-regulate their behavior. For example, a person’s workplace may provide workday opportunities to engage in PA (e.g., nearby park) or exposure to calorie-dense snacks (e.g., vending machines, shared food) not available at home. It is also possible that an association with PA and accessibility to nearby parks will be reduced by a high crime rate area. Similarly, passing fast food restaurants on the way to visit family may serve as trigger to deviate from usual dietary patterns. This study will allow us to capture these daily and momentary deviations in routine activity-space environments and identify their effects on daily and in-the-moment diet and PA behaviors.

Self-regulatory capacity encompasses trait and state factors that influence a person’s ability to make efforts to regulate their behavior such as mood states and internal drivers such as hunger (21, 22). Environmental supportiveness at any given time can affect behavior and make self-regulatory capacity and prepotency more or less influential (23). For example, when unhealthful foods are prevalent, greater self-regulatory capacity is required to engage in healthy behaviors and the effects of behavioral prepotency may be heightened. Thus, incorporation of personal trait and state factors will help us better understand for whom the environment matters and under what specific conditions.

The potential impact on public health is high. This study lays the groundwork for both policy and environmental changes, as well as just-in-time adaptive lifestyle and place-based interventions for mid-life adults. Lifestyle intervention effectiveness may be enhanced by supporting individuals in navigating their environment and momentary personal states (e.g., mood) and situational factors in real-time. A just-in-time adaptive intervention can continuously collect information on a person’s changing internal state and environmental context to provide to provide support in real-time, and in the right place thereby nudging healthier dietary and PA behaviors (31). By more precisely estimating environment-behavior associations using activity spaces and identifying environmental features most strongly associated with dietary and PA behaviors, our findings will help prioritize promising targets for environmental changes (e.g., trails and walking infrastructure) and policies (e.g., transit-oriented developments) to help make healthier choices, easier choices and ultimately promote healthy aging. Creating environments that provide opportunities and limit barriers or triggers for healthy dietary and PA behaviors may be especially important during mid-life, a period that is characterized by substantial time constraints and demands.

There are possible limitations to the current study that must be addressed. Given our intensive data collection, we were unable to recruit a probability sample and therefore propose a non-probability sample. However, several cohorts that yielded important information on adult health determinants are non-probability samples. Our between-person analyses of routine activity spaces and behaviors are cross-sectional but will provide the basis for future longer-term follow-up. We will maintain up-to-date contact information enabling a longitudinal study. In addition, not all instruments have been validated with Spanish-speaking Hispanic/Latinos. While not ideal, we chose to translate these measures into Spanish, rather than exclude this important subpopulation. While recruitment challenges are possible, our extensive experience and varied planned recruitment strategies allow us to overcome most difficulties. The successful subway advertising campaign may have inadvertently introduced mobility bias as subway users naturally tend to be more prone to daily walking during their commute. However, it should be considered that the population in this study was mid-life adults and the most common form of commuting in Cook county is mass transit and we collected mode of physical activity preference.

We will also conduct a series of sensitivity analyses to ensure robustness of findings (see data analysis section). The requirement of smart phone use and willingness to undergo intensive monitoring may also have introduced a technology bias, although it is unlikely that this would change the results considering the widespread use of cell phones in the mid-life population, and the population acceptance of daily monitoring as used in general society. It is possible that we may have missed a participant who performed swimming as exercise with the GPS and Actigraph measures, however, our surveys would capture this eventuality. Our study focused on environmental availability and visibility. Future studies should include affordability and quality as potentially relevant factors for diet behavior. Further, we did not directly measure self-control/inhibitory control and future studies should incorporate measures of self-control/inhibitory control for full assessment of the TST. Lastly, not all questionnaires have been validated in Spanish or among Hispanic/Latino populations possibly introducing bias. However, the translation was conduced by a certified medical translator, and all study interactions were conducted with bilingual Spanish speaking RAs.

This study of activity-space environment is poised to provide insight into between-person and within-person variations in diet and PA in mid-life and how environmental influences on behaviors are studied in mid-life and provide insights into the environment’s role in shaping usual, daily, and in-the-moment behaviors and for whom and under what conditions the environment matters. These findings will contribute to new targets for lifestyle and place-based interventions to improve health during mid-life and set the stage for better later-life health.

## 4 Ethics and dissemination

All study procedures followed the Declaration of Helsinki. We obtained approval from the local IRB (#2019-0630). Electronic informed consent was obtained from all participants prior their enrollment in the study. The results will be disseminated appropriately with respect for privacy and personal data protection. All electronic forms were kept in a secure electronic system accessible only to certain members of the study team. Laptops or tablets used to collect data were encrypted to protect the data. This data was transferred and stored on a secure server at the institution using a HIPPA approved secured Box Health cloud-based server. All electronic data files were encrypted and stored on restricted-access computer servers. The smartphone application, MetricWire, was approved by the IRB for its security and privacy protections. The information submitted through the smartphone application was encrypted on both the phone and on the server. The geocoding was conducted and stored on a secure server. For added security, the information is protected by a federal Certificate of Confidentiality. This Certificate means that information promised to protect cannot be obtained from the researchers by any means, legal or otherwise.
